# Optimized collaboration of the first and third signals endows robust activity to T cells within the immunocompetent tumor microenvironment

**DOI:** 10.1038/s41423-023-01050-9

**Published:** 2023-06-12

**Authors:** Jianshu Wei, Xin Lin, Weidong Han

**Affiliations:** 1 Changping Laboratory, Yard 28, Science Park Road, Changping District, 102206 Beijing, China; 2 Department of Basic Medical Sciences, Tsinghua University School of Medicine, 100084 Beijing, China; 3 Tsinghua-Peking Center for Life Sciences, 100084 Beijing, China; 4 Department of Biotherapy, the First Medical Center, Chinese PLA General Hospital, 100853 Beijing, China

**Keywords:** Immunosuppression, Cancer microenvironment

CAR T-cell therapy has shown remarkable potential in the treatment of hematologic malignancies, and these successes have greatly motivated research on treatment of solid tumors. Although numerous clinical trials have been actively carried out, exciting clinical responses are still sporadic and may be are transient for patients with solid tumors [1]. Such poor antitumor potency in solid tumors has been attributed to several aspects, including the heterogeneity of tumor-associated antigens (TAAs), poor trafficking of CAR T cells to the tumor site and inevitable off-tumor toxicities, as well as the immunosuppressive tumor microenvironment (TME).

To overcome these obstacles, many strategies have been explored, such as dual-targeting CARs designed to enhance the responsiveness of CAR T cells to heterogeneous solid tumors. This approach may improve the function of T cells by incorporating novel costimulatory molecules or modifying the signaling pathways downstream of CAR/TCR and reverting inhibitory signals or metabolic reprogramming to cope with the suppressive TME.

Optimal T-cell activation, proliferation and persistence require synergy between the first and third signals (cytokine support), but inflammatory cytokines are generally deficient in the TME. Accumulating evidence indicates that an adequate third signal for T cells may compensate for the inhibitory effects of the TME. For example, Freitas et al. recently applied genome-wide CRISPR knockout screens to identify Mediator complex sub12 (MED12) as a regulator of T-cell expansion. MED12 deletion enhanced antitumor activity and sustained the effect phenotype of CAR/TCR-T cells, which was attributed to improved IL2RA expression and IL-2 sensitivity [2].

To provide a sufficient third signal for T cells, systemic cytokine supplementation is the easiest strategy to implement, but clinical practice shows that it has prohibitive toxicity. Engineering T cells to locally release proinflammatory factors has the potential to overcome these toxicities. In the December 2022 issue of *Science*, Allen et al. reported engineering of T cells with a tumor-responsive autocrine IL-2 circuit. In this circuit, IL-2 expression is driven by tumor-specific synNotch receptors independent of CAR/TCR activation. Compared with other IL-2 delivery patterns, including systemic administration, constitutive IL-2 secretion, TCR/CAR activation-induced IL-2 production and paracrine IL-2 replenishment by gene-modified T cells, the conditionally initiated autocrine IL-2 circuit led to preeminent therapeutic outcomes in immunocompetent tumor models [3]. In the same issue of *Science*, the study of Li et al., which involved design a suite of orthogonal synthetic gene circuits controlled by small molecules, called the synZiFTR platform, to execute user-defined complex biological programs, is reported. The authors tested the efficacy of the synZiFTR platform in regulating the multifunctional output of CAR T cells in vivo using a xenograft liquid tumor model, and the results also demonstrated that an independent and sufficient supply of IL-2 is crucial for driving synergistic therapeutic responses in cooperation with CAR activation [4].

**Fig. 1:**
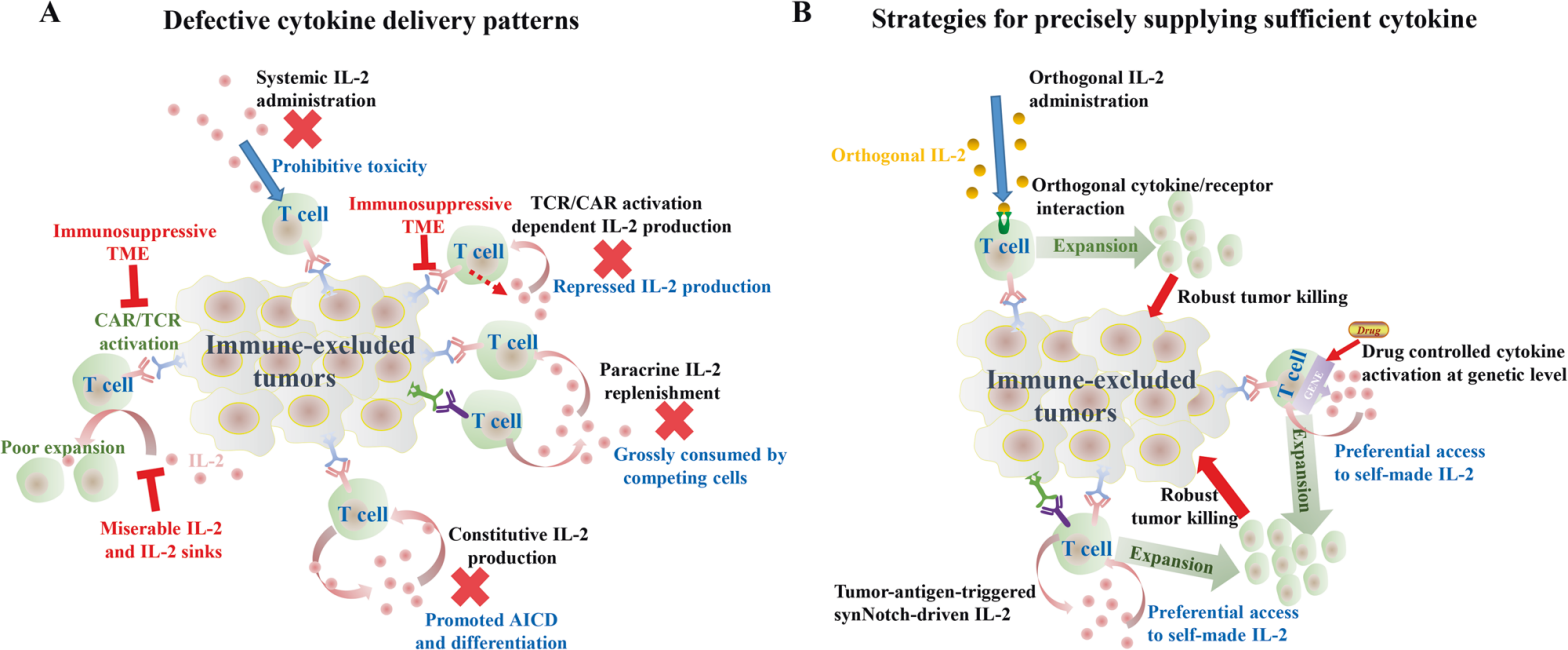
**A** Systemic administration, constitutive IL-2 secretion, TCR/CAR activation-induced IL-2 production and paracrine IL-2 replenishment by gene-modified T cells are defective in coping with the immunosuppression occurring in the TME. **B** Orthogonal cytokine/receptor interaction, tumor-antigen-triggered synNotch-driven IL-2 production and drug-induced controllable cytokine transcription at the genetic level can provide precise and sufficient third signals to T cells to evade key suppressive steps

These perspicacious studies suggest that supplying precise and sufficient third signals may be a general strategy to reconstitute the antitumor response of T cells within the immunosuppressive TME, and this requires three principles to be met, as shown in Fig. 1. First, IL-2 production should be conditional rather than constitutive. IL-2 has a biphasic effect on T cells, and persistent IL-2 exposure can cause significant activation-induced cell death (AICD) and promote T-cell differentiation. In addition, incessantly active genes risk payload silencing by some feedback regulation mechanisms. This may explain why immune-excluded tumor models are still resistant to CAR T cells with constitutive IL-2 expression, even though it is also autocrine. Second, CAR/TCR activation and IL-2 production should be independently induced. As a critical amplifier of T-cell activity, IL-2 is normally produced after T-cell activation. In a suppressive TME, T-cell activation is simultaneously limited by inhibition of CAR/TCR activation and low amounts of IL-2, which are grossly consumed by competing cells. It is virtually impossible for T cells to supply themselves with adequate IL-2 if the cytokine circuit depends on CAR/TCR activation. Establishing a bypass circuit for IL-2 production that is independent of CAR/TCR activation would evade the key suppressive steps, leading to the explosive T-cell expansion necessary for robust antitumor activity. Third, the host immune cells in solid tumors play a significant role as IL-2 sinks. Given their vast excess population, therapeutic T cells should have dominant IL-2 capture ability to ensure that the input third signal can meet the threshold required for full T-cell activation. This may explain why only the autocrine IL-2 delivery pattern endows T cells with robust activity within immune-excluded tumors, and the paracrine pattern does not. Autocrine cells have higher accessibility to their own IL-2. In addition, the IL-2 capture ability of autocrine cells could be further improved by expression of the high-affinity IL-2 receptor subunit CD25 triggered by CAR/TCR activation. As reported by Kalbasi et al., precise supply of a third signal for adoptively transferred T cells can be achieved through orthogonal IL-2 cytokines [5], in which T cells are modified by corresponding chimeric cytokine receptors.

In summary, these recent studies have collectively shown that optimized inflammatory cytokine delivery patterns that bypass the critical points of immune suppression are able to reconfigure the collaboration between the first and third signals, which may be a general strategy for improving the responsiveness of immunosuppressive solid tumors to T-cell therapies. Whether IL-2 is the ‘Chosen One’ requires further research. Kalbasi et al. found that a chimeric orthogonal IL-2 receptor gives rise to IL-9R signaling and improves the potency of T cells, suggesting the possibility of a better choice [5]. In addition, comprehensive intercomparisons between different strategies, such as the synNotch-driven autocrine cytokine circuit, small molecule-regulated synZiFTR platform, orthogonal cytokine system and other potential novel cytokine delivery methods, are needed to find the most favorable tool for conquering solid tumors.
